# Breast Reconstruction with DIEP Flap: The Learning Curve at a Breast Reconstruction Center and a Single-Surgeon Study

**DOI:** 10.3390/jcm12082894

**Published:** 2023-04-16

**Authors:** Charalampos Varnava, Philipp Wiebringhaus, Tobias Hirsch, Alexander Dermietzel, Maximilian Kueckelhaus

**Affiliations:** 1 Division of Plastic Surgery, Department of Trauma, Hand and Reconstructive Surgery, University Hospital Muenster, 48149 Muenster, Germany; 2 Department of Plastic, Reconstructive and Aesthetic Surgery, Hand Surgery, Fachklinik Hornheide, 48157 Muenster, Germany; 3 Institute of Musculoskeletal Medicine, University Hospital Muenster, 48149 Muenster, Germany

**Keywords:** breast reconstruction, DIEP, learning curve

## Abstract

Although microsurgical breast reconstruction represents a very interesting and rewarding field of plastic surgery, appropriate microsurgical training is not possible in every plastic surgery department. In this retrospective study, we present the learning curve of our plastic surgery department as a whole and of a single microsurgeon assessing breast reconstruction procedures with a deep inferior epigastric artery perforator (DIEP) flap between July 2018 and June 2021. The present study included 115 patients and 161 flaps. Cases were stratified into single DIEP/double DIEP groups and into early and late groups based on the flap order. Surgery times and postoperative complications were analyzed. Regarding the institution, the length of hospital stay was lower in the late group than in the early group (single 7.1 ± 1.8 vs. 6.3 ± 1.5 days, *p* = 0.019; double 8.5 ± 3.8 vs. 6.6 ± 1.4 days, *p* = 0.043). Apart from that, no statistically significant differences were found between the start and end of our study. In terms of the single surgeon, there was a significant improvement in the total surgery time (single 296.0 ± 78.7 vs. 227.5 ± 54.7 min, *p* = 0.018; double 448.0 ± 85.6 vs. 341.2 ± 43.1 min, *p* = 0.008), flap ischemia time (53.6 ± 15.1 vs. 40.9 ± 9.5 min, *p* = 0.007) and length of stay among the compared groups. There was no significant difference in flap loss rate or other complications between the early and late groups. Further performance of surgeries seemed to improve the surgeon’s skills as well as the overall experience of the medical institution.

## 1. Introduction

Breast reconstruction is a major subject in the field of plastic surgery and often aims to improve patients’ quality of life, regardless of their condition. Due to the advances in technology, microsurgeons have the opportunity to work with better devices, which facilitates the process. However, like in every other surgical field, experience plays an important role in the performance of such surgeries. A long learning curve may prove to be a barrier for some plastic surgeons.

Breast reconstruction is also a very demanding field of plastic surgery. It requires practical microsurgical skills, good spatial perception, extensive knowledge of anatomy and effective decision-making and creativity. All of these skills can potentially be improved through accumulating training and experience.

Not all plastic surgeons have the chance to be trained at a center with a high volume of microsurgical operations and breast reconstructions. As a result, some or many of them may never begin or give up quickly after the first complications occur.

The deep inferior epigastric perforator (DIEP) flap was first presented in 1989 by Koshima [1]. In breast reconstruction, Robert Allen was the one who introduced the DIEP flap as a reconstruction alternative in 1994 [2]. Since then, it has been established as the gold standard in free flap breast reconstruction.

Increased experience has been shown to improve complication rates [3,4,5]. Accurate judgment of adequate perforators also involves a learning curve [6].

This study aims to show the progress of an institution and of a single surgeon as they get more experienced with DIEP flap breast reconstruction. We investigate if the increase in microsurgical experience improves the quality of treatment.

## 2. Materials and Methods

This study was approved by the local ethics committee (Approval code: 2020-779-f-S. Approval Date: 25 April 2022).

We performed a retrospective study by reviewing the electronic medical records of our institution. We studied a total of 161 DIEP flaps in 115 patients performed over three years between July 2018 and June 2021 at our institution. This study included all patients undergoing either a unilateral breast reconstruction with a DIEP flap or a bilateral breast reconstruction with DIEP flaps during this period. The analysis of the calculated parameters was made for all the procedures as a whole and also separately, only for procedures performed by one of our surgeons (single-surgeon study). The procedures were performed by four senior surgeons: two with previous experience in breast reconstruction and two without. Therefore, the data presented reflect the learning curve of a newly established high-volume breast reconstruction center on the one hand and the learning curve of a single surgeon on the other. For the single-surgeon study, the cases in which a critical part of the surgery (flap raise, anastomosis, inset of the flap) was performed by another surgeon were excluded. During bilateral reconstructions, a single surgeon performed the elevation of both flaps while another surgeon simultaneously performed steps such as wound closure and recipient site preparation and closure.

For preoperative planning and perforator visualization, a computer tomographic angiography (CTA) was performed in every breast reconstruction with a DIEP flap.

All arterial anastomoses were hand-sutured, and all venous anastomoses were performed using a coupler device.

The cases were compared according to the total surgery time, ischemia time, hospital stay, flap loss and complications postoperatively. Wound dehiscence, seroma and infection were identified as donor-site complications.

**Figure 1:**
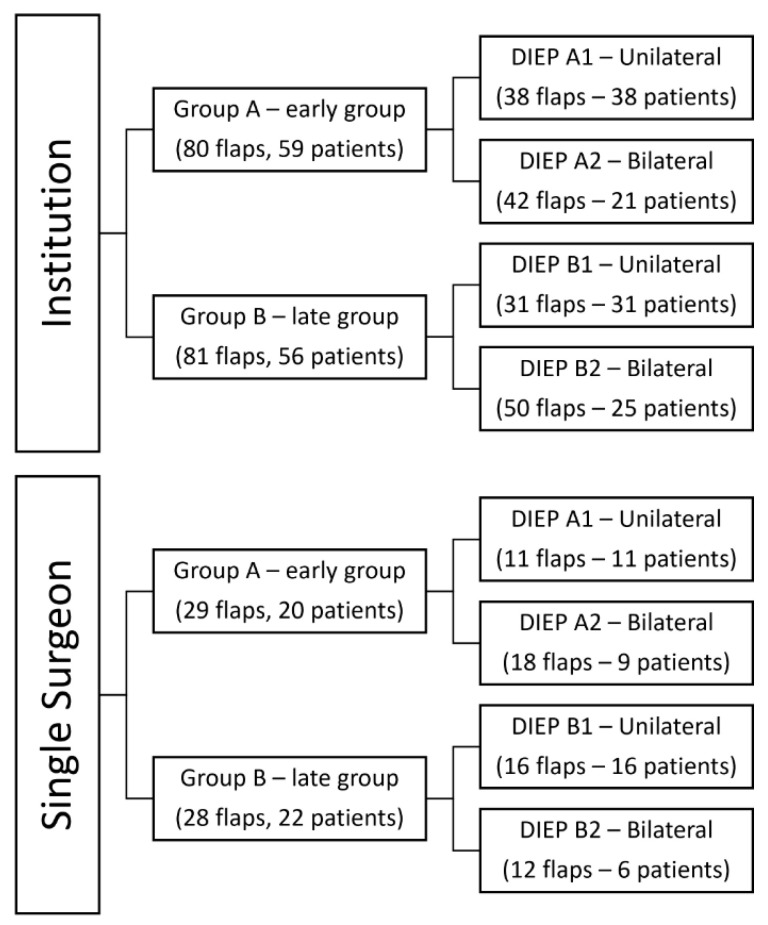
Group classification.

All performed DIEPs were split for purposes of analysis into two subgroups of 80 (early group—first half of flaps performed) and 81 flaps (late group—second half of flaps performed) and 59 and 56 patients, respectively. The single-surgeon DIEP group was also split into two subgroups of 29 and 28 flaps and 20 and 22 patients. From these subgroups, the single and double reconstructions were separated and analyzed (Figure 1).

For ischemia time and postoperative complication evaluation, both single and double DIEP reconstruction times were taken into account.

Data are reported as mean ± standard deviation and median. Scatter plots and box plots were used to visualize the change over time, with the x-axis showing the flap number in the specific group and the y-axis showing the measured parameter.

The retrieved data were documented in Excel. The statistical analysis was performed with Microsoft Excel v2104 (Redmond, WA, USA) and IBM SPSS Statistics v25 (Armonk, NY, USA). We tested for normality using the Kolmogorov–Smirnov test. The equality of variances was assessed using the Lavene test. The variables of each group were compared using the Pearson chi-square test, *t*-test, Fischer’s exact test and the Mann–Whitney test.

A *p*-value of <0.05 was considered statistically significant.

## 3. Results

### 3.1. Institution

Between July 2018 and June 2021, a total of 161 breast reconstructions with DIEP flaps were performed in our institution. Of the total number, 42.9% of the DIEP flaps (n = 69) were performed unilaterally, and 57.1% (n = 92) were performed bilaterally.

The mean patient age was 50.5 ± 10.2 years (median 51.9), and the mean BMI was 27.4 ± 4.7 kg/m^2^ (median 27).

In the first group, the mean age was 51.4 ± 10.6 years (median 52.4), and the mean BMI was 28.3 ± 4.7 kg/m^2^ (median 28). In the second group, the mean age was 49.6 ± 9.8 years (median 52.4), and the mean BMI was 26.4 ± 4.5 kg/m^2^ (median 26). P_age_ = 0.352, p_BMI_ = 0.029.

The average follow-up was 10.1 ± 5.7 months (median 11.3) for the team group.

**Figure 2:**
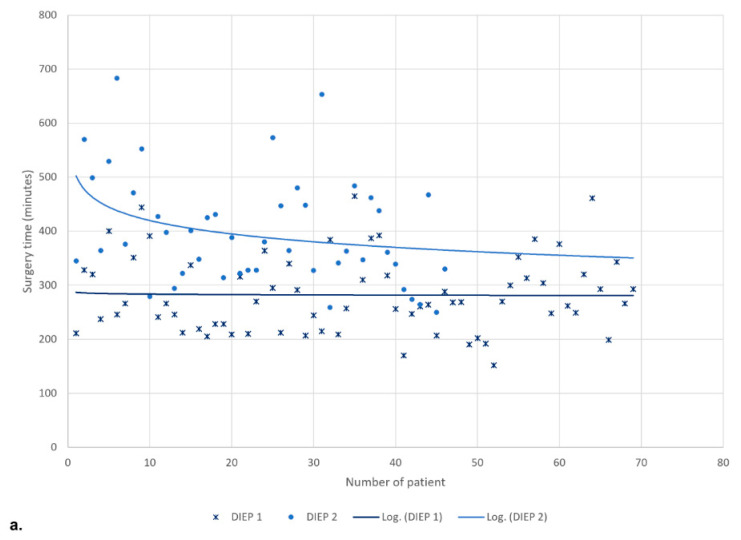

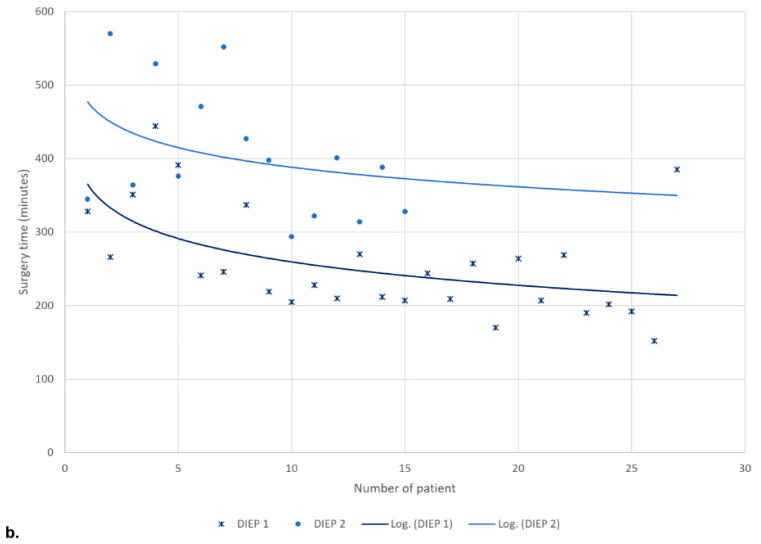
Institution (**a**) and Single-Surgeon Study (**b**)—Development of the overall surgery time with respect to the number of the flap-DIEP 1: single DIEP; DIEP 2: double DIEP.

The mean total surgery time of the single DIEP group was 282.2 ± 71.1 min (median 266), and of the double DIEP group was 398.6 ± 101.6 min (median 370) (Figure 2).

The first single DIEP group (A1) showed a mean total surgery time of 276.4 ± 67.4 min (median 251.5), and the second single DIEP group (B1) showed a mean of 287.8 ± 75.2 min (median 270) with *p* = 0.617.

**Figure 3:**
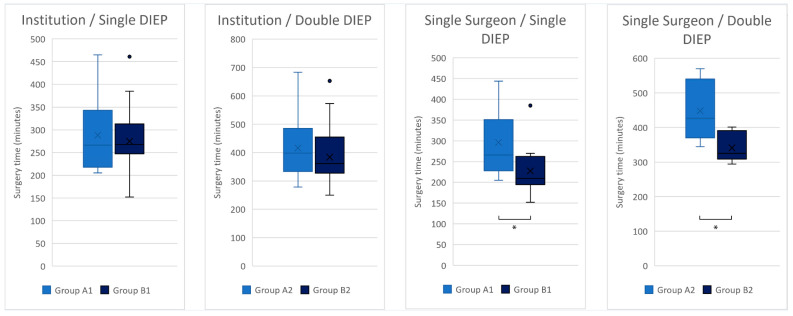
Institution and Single-Surgeon Study—Comparison of the surgery time of early and late groups for the single DIEP- and for the double DIEP-reconstructions. *: statistically significant.

The first double DIEP group (A2) showed a mean total surgery time of 408.4 ± 101.8 min (median 388), and the second double DIEP group (B2) showed a mean of 388.8 ± 102.7 min (median 363) with *p* = 0.290 (Figure 3).

**Figure 4:**
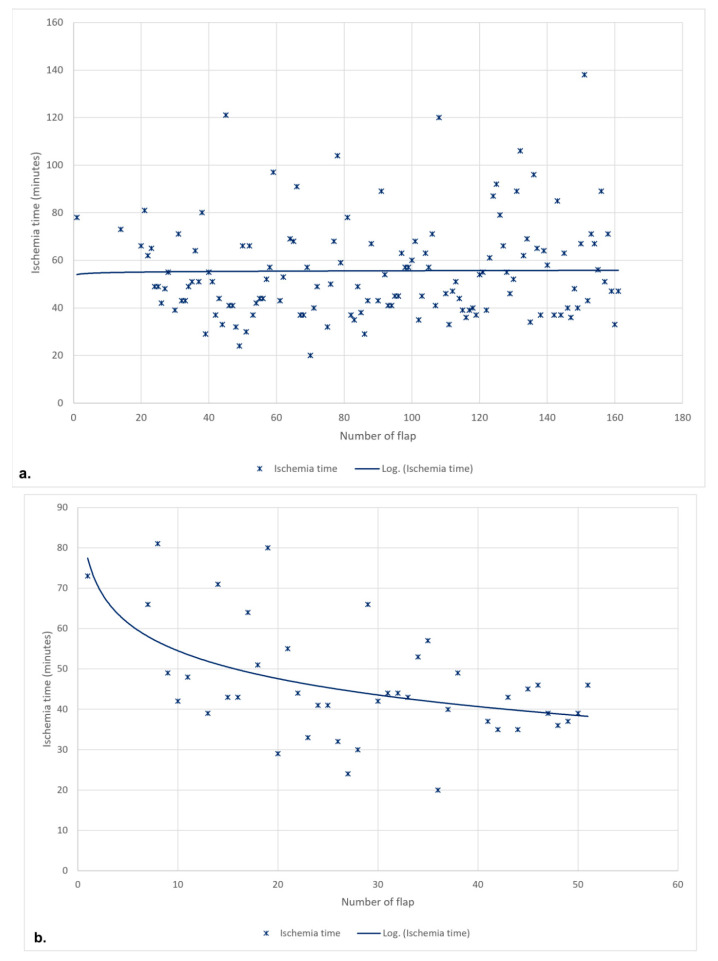
Institution (**a**) and Single-Surgeon Study (**b**)—Development of the flap ischemia time with respect to the number of the flap.

The mean ischemia time of the DIEP group was 55.5 ± 20.5 min (median 51). The mean ischemia time of the first total DIEP group was 54.0 ± 19.8 min (median 50), and of the second total DIEP group, 56.6 ± 21.1 min (median 51.5) with *p* = 0.640 (Figure 4).

In the first group, partial rib resection was performed to expose the internal mammary vessels in every case, whereas in the second group, a “rib-sparing” technique was used in 24.7% of the cases (n = 20). *p* < 0.001.

Six patients of the double DIEP group required an abdominal wall repair in the form of a plication due to a diastasis recti abdominis, three in the first group (14.3%) and three in the second (12.0%, *p* = 1.000). Five patients of the double DIEP group required an abdominal wall repair in the form of a mesh because of a fascia defect, three in the first group (14.3%) and two in the second (8.0%, *p* = 0.648). When a mesh was required, we performed an onlay mesh repair. No patients of the single DIEP group required an abdominal wall repair.

The mean length of stay for the single DIEP group was 6.7 ± 1.7 days (median 7), and for the double DIEP group was 7.5 ± 3.0 days (median 7).

The mean length of stay for the first single DIEP group was 7.1 ± 1.8 days (median 7) and 6.3 ± 1.5 days (median 6) for the second single DIEP group with *p* = 0.018. The mean length of stay for the first double DIEP group was 8.5 ± 3.8 days (median 8), and for the second double DIEP group, 6.6 ± 1.4 days (median 6) with *p* = 0.043.

From the first DIEP group with 80 flaps, six (7.5%) were taken back into the operating room for revision surgery because of an anastomosis problem (one with arterial thrombosis, four with venous thrombosis and one with intrinsic flap thrombosis). In the second DIEP group with 81 flaps, three were taken back into the operating room for revision (3.7%, one with arterial thrombosis, one with venous thrombosis and one with intrinsic flap thrombosis), *p* = 0.328. The first DIEP group showed 2.5% total flap loss (n = 2) and 6.3% partial flap loss (n = 5).

**Figure 5:**
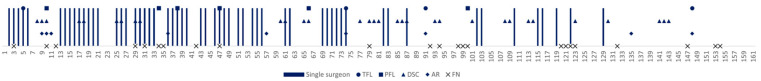
Timeline of the occurrence of events (y-axis) with respect to the number of consecutive DIEP-flap (x-axis)-TFL: total flap loss; PFL: partial flap loss; DSC: donor site complication; AR: anastomosis revision; FN: fat necrosis.

The second DIEP group showed 2.5% total flap loss (n = 2) and 1.2% partial flap loss (n = 1), p_tfl_ = 1.000 and p_pfl_ = 0.117. Fat necrosis was clinically present in 12.5% of the reconstructed breasts in the first group (n = 10) and in 16.0% in the second group (n = 13), *p* = 0.654). From the first group, 16.9% of the patients had a donor site complication (n = 10) and from the second group, 21.4% (n = 12, *p* = 0.638) (Table 1, Figure 5).

### 3.2. Single-Surgeon

Between July 2018 and June 2021, a total of 57 breast reconstructions with DIEP flaps were performed by the same plastic surgeon in our institution (27 single DIEP, 30 double DIEP and 42 patients). This plastic surgeon was responsible for the preoperative markings, flap design, flap raise, flap inset and anastomosis in these cases.

The mean patient age was 49.8 ± 11.5 years (median 51.2), and the mean BMI was 28.2 ± 4.4 kg/m^2^ (median 28).

In the first group, the mean age was 49.9 ± 10.3 years (median 50.3), and the mean BMI was 28.8 ± 4.9 kg/m^2^ (median 29). In the second group, the mean age was 49.6 ± 12.7 years (median 51.4), and the mean BMI was 27.6 ± 4.0 kg/m^2^ (median 27), p_age_ = 0.924, p_BMI_ = 0.363.

The average follow-up was 10.4 ± 6.0 months (median 11.5) for the single-surgeon group.

The mean total surgery time of the single DIEP group was 255.4 ± 72.7 min (median 241), and of the double DIEP group was 405.3 ± 88.2 min (median 388).

The operative time decreased as the number of cases increased in both the single-DIEP and the double-DIEP group. (Figure 2).

The first single DIEP group (A1) showed a mean total surgery time of 296.0 ± 78.7 min (median 266), and the second single DIEP group (B1) showed a mean of 227.5 ± 54.7 min (median 209.5) with *p* = 0.018.

The first double DIEP group (A2) showed a mean total surgery time of 448.0 ± 85.6 min (median 427), and the second double DIEP group (B2) showed a mean of 341.2 ± 43.1 min (median 325) with *p* = 0.008 (Figure 3).

The mean ischemia time of the DIEP group was 48.0 ± 15.3 min (median 44). The mean ischemia time of the first total DIEP group was 53.6 ± 15.1 min (median 49) and of the second total DIEP group, 40.9 ± 9.5 min (median 41) with *p* = 0.007 (Figure 4).

In the first group, a partial rib resection was performed to expose the internal mammary vessels in every case, whereas in the second group, a “rib-sparing” technique was used in one case (3.6%), *p* = 0.491.

One patient (16.6%) of the second double DIEP group and no patient from the first group required an abdominal wall repair in the form of a plication because of a diastasis recti abdominis (*p* = 0.400). One patient (11.1%) of the first double DIEP group and no patients from the second group required an abdominal wall repair in the form of a mesh because of a fascia defect (*p* = 1.000). When a mesh was required, we always performed an onlay mesh repair. No patients of the single DIEP group required any form of abdominal wall repair. The mean length of stay for the single DIEP group was 6.5 ± 1.7 days (median 6), and for the double DIEP group was 8.1 ± 4.2 days (median 7).

The mean length of stay was for the first single DIEP group 7.6 ± 1.8 days (median 7) and for the second single DIEP group 5.8 ± 1.2 days (median 6) with *p* = 0.007. The mean length of stay for the first double DIEP group was 8.9 ± 5.1 days (median 9), and for the second double DIEP group, 7.0 ± 2.1 days (median 6.5) with *p* = 0.438.

From the first DIEP group with 29 flaps, one patient (3.4%) was taken back into the operating room for revision surgery because of an anastomosis problem (arterial thrombosis). From the second DIEP group with 28 flaps, one patient was also taken back into the operating room for revision (3.6%, intrinsic flap thrombosis), *p* = 1.000. The first DIEP group showed 3.4% total flap loss (n = 1) and 3.4% partial flap loss (n = 1).

The second DIEP group showed 3.6% total flap loss (n = 1) and 3.6% partial flap loss (n = 1), p_tfl_ = 1.00 and p_pfl_ = 1.00. Fat necrosis was clinically present in 10.3% of the reconstructed breasts in the first group (n = 3) and 7.2% in the second group (n = 2), *p* = 0.669. From the first group, 15.0% of the patients had a donor site complication (n = 3) and from the second group, 9.1% (n = 2), *p* = 0.656 (Table 2, Figure 5).

## 4. Discussion

A modern breast reconstruction center is required to offer comprehensive options for breast reconstruction. One of these options is the microsurgical free flap reconstruction with the DIEP flap as the gold standard. The successful performance of this complex operation requires a certain amount of training and experience.

Through this study, we aimed to outline the impact of accumulated experience in DIEP breast reconstruction on total surgery time, flap ischemia time, time to ambulation and risk of complications postoperatively.

Ischemia time is an independent risk factor for microvascular complications in breast reconstruction using a DIEP flap [7]. A significant association between ischemia time and fat necrosis rate has already been shown in previous studies [8,9,10], although some suggest the existence of a threshold after which the tissue damage becomes irreversible [7,11]. According to Lee et al. [10], efforts to reduce ischemia time in breast reconstruction using DIEP flaps are desirable in order to decrease the risk of fat necrosis. We were able to show that for a single surgeon, the accumulation of experience reduces the ischemic time.

Some parameters like flap weight, number of perforators, venous drainage, island flap usage, preoperative CTA and the learning curve appear to play a role in determining the operative time [12,13,14,15]. Interestingly, Santanelli di Pompeo et al. recently introduced an algorithm and demonstrated that harvesting the DIEP flap in a “free style technique” without depending on a CTA did not increase operative times or complication rates [16]. This technique is based on clinical judgment, which also requires a specific level of experience. According to Laporta et al., the learning curve appeared to be one of the most important variables able to reduce the total surgery time [12].

Bodin et al. demonstrated a significant DIEP learning curve regarding three parameters: surgical time, surgical revision rate and postoperative hospital stay [17]. Cubitt et al. showed a DIEP learning curve that was reflected in declining complications [18].

Selber et al. reported a learning curve and an increase in microsurgical skills assessed with the Structured Assessment of Microsurgery Skills (SAMS) [19]. In other surgical fields, it was also shown that a certain number of cases were required to reach technical proficiency [20,21,22,23,24,25,26,27,28,29,30,31,32].

Our findings seem to agree with this suggestion. We demonstrate that for the single surgeon, every analyzed intraoperative parameter and also the length of hospital stay was improved the more experience the surgeon gained. Meanwhile, the revision and flap loss rate remained the same. Very high case numbers are required to capture a significant change in flap survival rates throughout the learning curve. This is attributed to the generally very low flap loss incidence.

Santanelli et al. showed no significant association between the learning curve and partial flap necrosis in a series of 247 cases over seven years. However, the experience and confidence with the procedure resulted as protective factors confirming a progression of learning from the second to the seventh year [33].

Acosta et al. showed a significant success rate increase in unilateral DIEP breast reconstruction from 90.7% in 2000 to 98.2% in 2008. Moreover, the same study showed a dramatic decrease in the mean surgery time over nine years from 7 h and 18 min to 4 h and 8 min [34].

We were able to show in our single-surgeon study group that the mean operating time was reduced by a mean time of 68.5 min for the single DIEP and 106.8 min for the double DIEP over three years.

According to Beudeker et al., a novice plastic surgeon has similar results in complication rates compared to a center of excellence. However, a learning curve in terms of operating time is expected [35].

Postoperative complications consume considerable healthcare resources.

Also, in terms of cost-effectiveness, a reduced operative time and length of hospital stay are desirable [35,36,37]. Surgical site infections are associated with central-line associated bloodstream infections and ventilator-associated pneumonia with high costs [38].

Previous studies showed a significant association between extended operative time and surgical site infection [39,40,41,42,43]. Operative time is an independent risk factor for surgical site infection. This may be due to longer wound exposition to the environment resulting in a higher risk of bacterial contamination [39]. A study of 104,632 cases of 35 procedures of different surgical disciplines also demonstrated a correlation between operation times and complications [44].

According to Fischer et al.’s analysis of 16,063 cases of breast reconstruction, prolonged operative time is a risk factor for surgical and medical complications postoperatively [45]. Thorarinnson et al. demonstrated that a long duration of surgery could explain a substantial risk increase for early complications after breast reconstruction [46]. Korol et al. suggested that patients with longer hospital stays are also at an increased risk for surgical site infections [41]. Various other studies showed that increased operative time was a risk factor predictive of a related reoperation, development of a surgical site infection, prolonged length of stay and postoperative pulmonary complications [47,48,49].

In the single-surgeon group, we showed a statistically significant decrease in total surgery time.

The choice of different recipient vessels, such as axillary vessels, may reduce the operating time [50]. However, in our study, the internal mammary vessels were routinely used.

Parrett et al. described the rib-preservation technique for internal mammary vessel exposure in microsurgical breast reconstruction [51]. Other authors have highlighted possible drawbacks of sacrificing a rib, such as a contour deformity or intercostal neuralgia [52,53]. Throughout our development, we were able to significantly increase the number of rib-sparing cases from 0 (0%) in the first group of flaps to 20 (24.7%) in the second group. Although rib preservation can potentially influence the total surgery time and postoperative complications, this was not demonstrated in our study.

Surgeon and staff experience, fatigue, preoperative planning, equipment, and patient selection are also parameters potentially impacting operating time. Through this study, we show that parameters like total surgery time, flap ischemia time and length of hospital stay decrease as experience increases, whereas the revision rate and flap loss rate remained low. The number of cases with fat necrosis and donor site complications also did not show a statistically significant difference.

Despite the length of stay, there was little difference in most parameters between the early and late institution groups. In our opinion, this can be explained by the fact that operations were initially performed only by the most experienced surgeons in microsurgery but later also by microsurgeons without the same extent of experience in free flap breast reconstruction. However, the total operation time, the hospital stay, the revision rate and the flap loss rate remained low, which is an important aspect for a newly established breast reconstruction center.

Another influencing factor may be the numerous other microsurgical operations and free flap reconstructions (e.g., extremity reconstructions) performed by our surgeons during the time of the study, which also contributes to the accumulation of experience relevant to breast reconstruction. Although true, this is also a general rule which applies to every part of the surgical life of a surgeon, so we accordingly did not take this into account for our calculations. 

One limitation was the retrospective nature of the study. The surgeon for whom the data was analyzed for the single-surgeon study was a senior surgeon with microsurgical experience in other fields of plastic surgery. The sample size for the single-surgeon study is relatively small, with a short follow-up period. Furthermore, the number and type of perforators harvested, as well as the flap weight, which influence the total surgery time, were not always documented. There is also a performance bias as some of the operating surgeons had performed this type of surgery before, as mentioned earlier. A standardized assessment of microsurgical skills to evaluate skill acquisition, which was not used during the study, may have further enriched the conclusions. Another very important factor for the measure of success in breast reconstruction is the patient-reported outcomes, which were not included in this study.

## 5. Conclusions

Although it is commonly anticipated that experience plays a major role in medicine and specifically in plastic surgery, our data further support the expectations in a single-surgeon study. Experience is a key factor in improving the quality of treatment in breast reconstruction. With an increase in experience comes a decrease in operation time and length of hospital stay. Although microsurgery and breast reconstruction are difficult to master, persistence and further performance of surgeries seem to improve the surgeon’s skills in favor of the patients as well as the overall experience of the medical institution.

## Figures and Tables

**Table 1:** Institution (Mean ± SD).

	Group A (%)	Group B (%)	*p*
No. of flaps			
Total	80	81	
Unilateral	38	31	
Bilateral	42	50	
No. of patients			
Total	59	56	
Unilateral	38	31	
Bilateral	21	25	
Follow-up (months)			
	11.6 ± 6.2	8.6 ± 4.7	0.007 *
Age (years)			
	51.4 ± 10.6	49.6 ± 9.8	0.352
BMI (Kg/m^2^)			
	28.3 ± 4.7	26.4 ± 4.5	0.029 *
Total surgery time (min)			
Single DIEP	276.4 ± 67.4	287.8 ± 75.2	0.617
Double DIEP	408.4 ± 101.8	388.8 ± 102.7	0.290
Flap ischemia time (min)			
	54.0 ± 19.8	56.6 ± 21.1	0.640
Rib preservation			
	0 (0)	20 (24.7)	<0.001 *
Length of stay (d)			
Single DIEP	7.1 ± 1.8	6.3 ± 1.5	0.019 *
Double DIEP	8.5 ± 3.8	6.6 ± 1.4	0.043 *
Anastomosis revision			
	6 (7.5)	3 (3.7)	0.328
Total flap loss			
	2 (2.5)	2 (2.5)	1.000
Partial flap loss			
	5 (6.3)	1 (1.2)	0.117
Fat necrosis			
	10 (12.5)	13 (16.0)	0.654
Donor site complication			
	10 (16.9)	12 (21.4)	0.638

* Statistically significant; SD: standard deviation.

**Table 2:** Single surgeon (Mean ± SD).

	Group A (%)	Group B (%)	*p*
No. of flaps			
Total	29	28	
Unilateral	11	16	
Bilateral	18	12	
No. of patients			
Total	20	22	
Unilateral	11	16	
Bilateral	9	6	
Follow-up (months)			
	11.2 ± 6.2	9.7 ± 5.9	0.432
Age (years)			
	49.9 ± 10.3	49.6 ± 12.7	0.924
BMI (Kg/m^2^)			
	28.8 ± 4.9	27.6 ± 4.0	0.363
Total surgery time (min)			
Single DIEP	296.0 ± 78.7	227.5 ± 54.7	0.018 *
Double DIEP	448.0 ± 85.6	341.2 ± 43.1	0.008 *
Flap ischemia time (min)			
	53.6 ± 15.1	40.9 ± 9.5	0.007 *
Rib preservation			
	0 (0)	1 (3.6)	0.491
Length of stay (d)			
Single DIEP	7.6 ± 1.8	5.8 ± 1.2	0.007 *
Double DIEP	8.9 ± 5.1	7.0 ± 2.1	0.438
Anastomosis revision			
	1 (3.4)	1 (3.6)	1.000
Total flap loss			
	1 (3.4)	1 (3.6)	1.000
Partial flap loss			
	1 (3.6)	1 (3.6)	1.000
Fat necrosis			
	3 (10.3)	2 (7.2)	0.669
Donor site complication			
	3 (15.0)	2 (9.1)	0.656

* Statistically significant; SD: standard deviation.

## Data Availability

Data available on request.
